# Obsessive‐compulsive disorder: Etiology, neuropathology, and cognitive dysfunction

**DOI:** 10.1002/brb3.3000

**Published:** 2023-05-03

**Authors:** Baland Jalal, Samuel R. Chamberlain, Barbara J. Sahakian

**Affiliations:** ^1^ Department of Psychiatry University of Cambridge School of Clinical Medicine Cambridge UK; ^2^ Behavioural and Clinical Neuroscience Institute, Department of Psychology University of Cambridge Cambridge UK; ^3^ Faculty of Medicine, Department of Psychiatry University of Southampton Southampton UK; ^4^ Specialist Clinic for Impulsive and Compulsive Disorders, and the Southern Gambling Service Southern Health NHS Foundation Trust Southampton UK

**Keywords:** cognitive function, cognitive models, etiology, learning‐based models, neuropathology, obsessive‐compulsive disorder

## Abstract

**Background:**

This review provides an overview of obsessive‐compulsive disorder (OCD) symptoms, including the four partially distinct subtypes of the disorder, current diagnostic criteria, and common comorbidities. Critically, it focuses on the etiology of OCD, including its underlying neuropathology, and examines cognitive dysfunction in OCD.

**Methods:**

This review study was conducted by library method.

**Results:**

We show how dysfunction in cortico‐striato‐thalamo‐cortical (CSTC) circuits may underpin symptoms; and shed light on the putative neurochemistry within these loops such as the role of serotonin, dopamine, and glutamate systems. We also show how OCD is characterized by cognitive dysfunction including problems in cognitive flexibility, visuospatial memory, response inhibition, and goal‐directed behavior, linked to aberrant activity within CSTC circuits.

**Conclusions:**

In brief, research questions we shed light on include (1) what are the symptoms in OCD; (2) what is the etiology of the disorder and do existing models explain OCD; and (3) what are key cognitive deficits in OCD and do these improve with treatment?

## Graphical abstract

This review provides an overview of obsessive‐compulsive disorder (OCD) symptoms, etiology, and examines cognitive dysfunction in OCD.

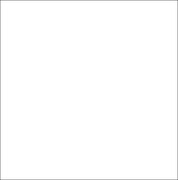

## 1 INTRODUCTION

Obsessive‐compulsive disorder (OCD) is a neuropsychiatric condition characterized by unwanted obsessions and/or compulsions. OCD affects 1–3% of the general population making it a common psychiatric disorder (Fawcett et al., 2020; Jalal et al., 2020; National Institute of Mental Health, 2017; Robins et al., 1984; Ruscio et al., 2010). The condition is highly debilitating, resulting in great suffering for patients worldwide (Stein, 2002). While the etiology of OCD is not fully understood various disease models have been proposed. These range from neurobiological theories implicating cortico‐striato‐thalamo‐cortical circuits in the etiology of the disorder (Chamberlain et al., 2005; Graybiel & Rauch, 2000; Milad & Rauch, 2012; see also, van den Heuvel et al., 2016); to learning‐based models stressing fear acquisition and extinction (Foa, 2010; McGuire et al., 2016; Shafran, 2005; Taylor et al., 2007; Tracy et al., 1999); and related cognitive models highlighting faulty beliefs (i.e., pertaining to obsessional anxiety) in the pathogenesis of OCD (Abramowitz, 2006, 2005). In addition to clinical symptoms, research suggests specific cognitive deficits in OCD. These affect domains such as cognitive flexibility, visuospatial memory, response inhibition, and goal‐directed behavior, and are linked to aberrant activity within CSTC circuits (Chamberlain et al., 2005, 2006a, 2021; Gillan et al., 2011; Lipszyc & Schachar, 2010; Morein‐Zamir et al., 2010).

This review provides an overview of the symptoms in OCD, as well as contemporary diagnostic criteria and common comorbidities. A key aim is to highlight the etiology of the disorder, including OCD neuropathology, for example, how dysfunction in CSTC circuits may underpin symptoms, and the putative neurochemistry within CSTC loops such as the role of serotonin, dopamine, and glutamate systems. Additionally, we will review learning‐based and cognitive models of OCD, highlighting their strengths and weaknesses. Another aim is to shed light on cognitive deficits in OCD. In sum, research questions include (1) what are the symptoms in OCD; (2) what is the etiology of the disorder and do existing models explain OCD; and (3) what are key cognitive deficits in OCD and do these improve with treatment.

## 2 METHODS

This narrative review was conducted by library method. A reviewer undertook a search on well‐known electronic databases such as Google Scholar. The search comprised of synonyms often associated with OCD, and the stated research questions such as “OCD and brain function.” Moreover, searches were conducted through reference lists of retrieved articles and pertinent reviews. The latest literature searches for this article were conducted in December 2022. The review was comprehensive, for example, included original empirical research articles, review articles, meta‐analyses and book chapters. Studies of subjects who were subclinical or nonclinical or with an unconfirmed diagnosis, as well as animal studies, were not excluded but always explicitly stated in the text. All articles were written in English and had been published (e.g., in a peer‐reviewed journal or edited book).

### 2.1 Symptoms, diagnosis, and comorbidities

OCD symptoms can be classified into partially distinct but also overlapping subtypes based on their presentation: (1) contamination fears and compulsive cleaning; (2) obsessive thoughts about causing harm and compulsive checking rituals; (3) obsessions with symmetry and compulsive ordering; (4) obsessions with collecting useless objects and compulsive hoarding (Stein, 2002). A purely obsessional subtype has been proposed with mixed empirical support, characterized by unwanted thoughts about sex, violence, and blasphemy (Baer, 1994; Williams et al., 2011). Moreover, according to a recent view, hoarding compulsivity, which affects around 30% of sufferers, may be etiologically distinct from other subtypes of OCD. Accordingly, hoarding disorder was categorized in the latest edition of the Diagnostic and Statistical Manual of Mental Health Disorders (DSM‐5) as a condition in its own right, within the OCD and related disorders category (American Psychiatric Association, 2013; Ayers et al., 2017; Chen et al., 2017; Samuels et al., 2002). Interestingly, one factor‐analysis of OCD symptoms in a large child and adolescent sample did not find hoarding to separate from other key symptoms (e.g., obsessions and/or compulsions about harm/sex, symmetry, contamination/cleaning), suggesting that hoarding symptomatology perhaps does not become distinct from other factors (i.e., OCD subtypes) until postadolescence (Højgaard et al., 2017).

The diagnosis of OCD relies on clinical assessment. Specific diagnostic criteria according to the DSM‐5 include having obsessions and/or compulsions that are time‐consuming (e.g., take an hour or more per day), and cause considerable distress or interfere with everyday activities (e.g., social and occupational functioning). These symptoms must not be a consequence of other mental or physical health disorders (Hirschtritt et al., 2017). The fact that the DSM stipulates that having either obsessions *or* compulsions is sufficient for a diagnosis (not both) suggests that these can theoretically occur separately, although in most cases, both types of symptom are present. The functional/causal relationship between obsessions and compulsions is not always clear—despite a traditional view that obsessions lead to compulsions, this may not always be the case (Abramowitz & Jacoby, 2014; Gillan et al., 2015; Gillan et al., 2016; Robbins et al., 2019).

The DSM‐5 includes a specifier about the level of insight into obsessions and compulsions (i.e., awareness of their senselessness); it recognizes that OCD patients have varying degrees of insight, with some patients having *good* or *fair insight* whereas others *poor insight* and even *absent insight/delusional beliefs* (Abramowitz & Jacoby, 2015). This specifier may enhance diagnostic classification as it, for example, stresses that some OCD patients (at certain points of the illness) can have outright delusional beliefs, not attributable to schizophrenia etc. (Abramowitz & Jacoby, 2014). Interestingly, OCD patients’ level of insight may differ for specific symptoms (they might have ample insight into certain obsessions and lack insight into others); and levels of insight can shift over time (Abramowitz & Jacoby, 2015; for recent studies on insight in OCD, see Manarte et al., 2021a, 2021b).

Notably, while overarching OCD diagnostic criteria—vis‐à‐vis obsessions and compulsions—have largely remained the same since the 1980s, in DSM‐5 OCD is no longer classified as an anxiety disorder, but an “obsessive‐compulsive and related disorder” (OCRD). This category includes, as noted, hoarding disorder, and also, body dysmorphic disorder (BDD), trichotillomania (hair‐pulling disorder) and excoriation (skin‐picking) disorder, etc. (Abramowitz & Jacoby, 2015). The OCRDs clustering arguably reflects a better understanding of the neurobiological substrates underlying these disorders of compulsivity (Marras et al., 2016). Akin to the DSM‐5, the latest edition of the World Health Organization's International Classification of Diseases (ICD‐11) has likewise shifted OCD into a new OCRD category (previously a “Neurotic, Stress‐related, and Somatoform Disorder”). In spite of this new classification, the DSM‐5 and ICD‐11 still recognize the strong link between the OCRDs and the anxiety disorders; evident by the fact that the two chapters appear next to each other in both classification systems (Reddy et al., 2018). All in all, this new regrouping may tentatively pave the way for more empirically grounded psychiatric classification (e.g., nosology underpinned by neuroscience and genetics); and have useful clinical applicability (Marras et al., 2016; Stein, 2019). As the OCRDs tend to cooccur, this diagnostic approach may lead to early detection; once one condition is identified, the clinician might further inquire about other OCRDs (Fineberg et al., 2018). This is important given that many of the OCRDs are frequently overlooked, with many patients not receiving treatment opportunities.

OCD often cooccurs with other psychiatric disorders, with lifetime comorbidity rates as high as 50–60% (Denys et al., 2004). Common cooccurring disorders include depression (66%), specific phobia (22%), social anxiety disorder (18%), eating disorder (17%), alcohol dependence (i.e., “alcohol use disorder” per DSM‐5) (14%), and panic disorder (12%) (Fineberg et al., 2007). OC spectrum disorders also commonly cooccur with OCD but are rarely measured at all in national epidemiological prevalence studies that do include OCD. In spite of the frequent comorbidity between OCD and the anxiety disorders, one study found that OCD patients are more likely to report lifetime OCD spectrum disorders (e.g., trichotillomania, skin‐picking disorder, and tic‐related disorders) relative to those with social anxiety and panic disorder, broadly in line with the new DSM‐5 OCRD category (Richter et al., 2003; see also Fineberg et al., 2007).

### 2.2 Etiology: neuropathology and neurochemistry of OCD

#### 2.2.1 CSTS circuits and OCD neuropathology

Research has implicated parallel CSTC circuits in the pathophysiology of OCD (Chamberlain et al., 2005; Graybiel & Rauch, 2000; Milad & Rauch, 2012; see also, van den Heuvel et al., 2016), that is, responsible for reward‐ and motivational‐related processes, executive function, motor and response inhibition, and habit‐based behavior (Fineberg et al., 2018). CSTC circuits project from frontal‐cortical regions to the striatum, and then onward to thalamic sites, from where they loop back to the cortex (Milad & Rauch, 2012). The direct and indirect pathways within these circuits have opposing net effects on the thalamus, resulting in either increased (i.e., direct pathway) or decreased cortical excitation (i.e., indirect direct pathway). An imbalance between these two pathways is thought to contribute to OCD pathology, that is, overactivity in the direct pathway (critical for initiation and suppression of behavior) creating a positive feedback loop resulting in CSTC circuit hyperactivity (Saxena et al., 2001; see also Maia et al., 2008; Ting & Feng, 2011) (Vahabzadeh & McDougle, 2014; van den Heuvel et al., 2016).

Studies have revealed structural abnormalities in CSTC circuits, particularly those implicating the orbitofrontal cortex (OFC), that is, reduced volume of this region (e.g., Atmaca et al., 2007), and, less consistently, reduced volume of the striatum in OCD (e.g., Robinson et al., 1995; for a review, see Menzies et al., 2008). Moreover, research has shown enhanced activation of these CSTC circuits in OCD patients, in the resting state and following symptom provocation. In particular, OCD symptom provocation is linked to increased activation of the OFC and striatum, including the caudate (Saxena et al., 1999; for a review see, Robbins et al., 2019; for a meta‐analysis, see Whiteside et al., 2004). Conversely, resting‐state CSTC hyperactivation may normalize following pharmacological and psychological treatment (Baxter et al., 1992; Saxena et al., 1999; see also, Fineberg et al., 2018). Interestingly, selectively disrupting these loops using surgical intervention (e.g., anterior cingulotomy) may lead to reduced volume of the caudate nucleus (Rauch et al., 2000), and improvement in OCD symptomatology (Fineberg et al., 2018; Jenike, 1998; Stein, 2002). And repeated stimulation (over several days) of the OFC and ventromedial striatum (VMS) within CSTC circuits using optogenetics triggers OCD‐like grooming behaviors in mice (Ahmari et al., 2013).

Over the years several disease models have been proposed and revised, implicating specific CSTC circuits in OCD pathology (Chamberlain et al., 2005; Graybiel & Rauch, 2000; Menzies et al., 2008; Milad & Rauch, 2012; van den Heuvel et al., 2016). One model stresses the OFC, grounded in extensive research implicating its role in OCD, as noted above (Menzies et al., 2008; Milad & Rauch, 2012). This is the so‐called “orbitofrontal striatal model” (i.e., the circuit projecting from the OFC to the striatum, onward to the thalamus before looping back to the OFC; Menzies et al., 2008). A related model proposed dysfunction in three functionally distinct CSTC circuits in OCD (Milad & Rauch, 2012): (1) “the affective circuit” involved in emotion and reward‐associated processing; it projects from the anterior cingulate cortex (ACC) and the ventromedial prefrontal cortex (vmPFC) to the nucleus accumbens, and then on to the thalamus, before looping back to the ACC and vmPFC. (2) The “dorsal cognitive circuit” is pertinent to executive function including working memory; projecting from the dorsolateral prefrontal cortex (dlPFC) to the caudate nucleus, then the thalamus before looping back. (3) The “ventral cognitive circuit” is responsible for motor and response inhibition, starting from the anterolateral OFC, then going to the putamen, thalamus, and then back to the original cortical region. Additionally, in light of the recent shift in thinking about OCD as a “disorder of compulsivity,” a (4) “sensorimotor circuit” has been proposed, projecting from premotor cortical regions to the putamen, and then thalamus, and back to the cortex. This circuit is involved in habit (automatic stimulus‐response)‐based behavior thought to contribute to compulsivity (van den Heuvel et al., 2016; see also Fineberg et al., 2018). Taken together, dysfunction in CSTC circuits, important for emotion and reward‐associated processing, executive function, motor and response inhibition, and habit formation may contribute to propensity for inflexible thoughts and behaviors (e.g., inhibitory deficits) and thereby OCD symptoms (Chamberlain et al., 2005).

Notably, early on these CSTC circuits were considered to be fully closed (i.e., segregated). But a more recent understanding (including of their structural overlap) points to considerable functional interactions between them (Milad & Rauch, 2012; van den Heuvel et al., 2016). For instance, cross‐talk between the “affective circuit” and “sensorimotor circuit” is crucial for the formation of habitual behaviors and associated compulsivity (on CSTC between‐circuit interplay, see Robbins et al., 2012; van den Heuvel et al., 2016). Likewise, the earliest CSTC account did not factor in the affective function of limbic centers, including the amygdala implicated in fear and anxiety (Milad & Rauch, 2012). This is relevant given the strong anatomical link between the amygdala and striatum (with amygdaloid projections to large areas of the striatum; on cortico‐amygdala‐striatal circuits see, Cho et al., 2013) (van den Heuvel et al., 2016). Fittingly, however, limbic regions, including the basolateral amygdala and hippocampus well‐connected to the OFC, were incorporated in a later orbitofrontal striatal model, that is, more aligned with our contemporary understanding of the involvement of these limbic centers in emotional states (Menzies et al., 2008; on the amygdala in OCD, see van den Heuvel et al., 2016; for a recent study showing distinct alterations in amygdala subregional functional connectivity in early‐ versus late‐onset OCD, see Cao et al., 2021). Consistent with this expanded orbitofrontal striatal model, a worldwide meta‐analysis of 1830 OCD patients and 1759 control subjects found smaller hippocampal volumes in patients (Boedhoe et al., 2017; such hippocampal volume might help explain visuospatial deficits in OCD discussed below).

Finally, the initial CSTC account did not make any distinction between OFC subareas. However, it is now established that the lateral and medial OFC have different functions (vis‐à‐vis processing of affective‐, reward‐, and fear‐related information), that is, being either hyperactive or hypoactive in OCD depending on the specific experimental context (Robbins et al., 2019; one model per se proposes a hyperlateral versus a hypomedial OFC in OCD; see Milad & Rauch, 2012). Of note, reduced functional connectivity between the lateral OFC and dorsal caudate is linked to cognitive inflexibility (that is, greater set shifting errors) in patients with OCD (Vaghi et al., 2017b). Likewise, as mentioned, OCD patients and their OCD‐free first‐degree relatives show hypoactivation of the lateral OFC during a reversal learning task (Chamberlain et al., 2008). In contrast, the medial OFC appears to be important for affective processing (see Tyagi et al., 2019), for example, associated with excess activation during early fear learning (conditioning) in patients with OCD (i.e., indicative of safety learning impairments) (Apergis‐Schoute et al., 2017). Likewise, reduced functional connectivity between the basolateral amygdala and medial OFC has been reported in OCD, predicting improved cognitive behavior therapy (CBT) intervention (Fullana et al., 2017).

Relatedly, one study examined the effectiveness of DBS of the ventral capsule/ventral striatal (VC/VS) or anteromedial subthalamic nucleus (amSTN) (and also combined stimulation of both sites) (12 weeks per stimulation period) across a group of refractory OCD patients. One key aim was to specifically assess the effects of DBS on mood and cognitive flexibility. Both VC/VS and amSTN DBS improved OCD symptoms to the same degree indicating comparable efficacy. Yet, interestingly, DBS of the amSTN, unlike the VC/VS site, improved cognitive flexibility. VC/VS DBS, on the other hand, had a more pronounced impact on mood (Tyagi et al., 2019). Moreover, imaging (tractography) showed that DBS at each respective location linked to separate neural networks: amSTN DBS chiefly to the lateral OFC (and also the dlPFC and dorsal anterior cingulate cortex); and VC DBS the medial OFC (and regions like the thalamus and amygdala). Taken together, this suggests that separate neural systems underlie unique OCD symptomatology that may improve in response to treatment. These findings are in keeping with clinical observation and research showing that selective serotonin reuptake inhibitors (SSRIs) ameliorate both OCD symptoms and mood (Tyagi et al., 2019), whereas serotonin manipulations do not typically impact EDS stage performance (Rogers et al., 1999; for an animal study see, Clarke et al., 2005; see also Vaghi et al., 2017b).

Likewise, research has shown that repetitive transcranial magnetic stimulation (rTMS) over the right orbitofrontal cortex in OCD patients (two 1‐week treatment periods) led to a reduction in symptoms versus sham‐stimulation. Follow‐up PET scan contrasts showed that active stimulation was linked to a bilateral decrease in the metabolism of the OFC, stressing the role of the OFC in OCD (Nauczyciel et al., 2014). Relatedly, another noninvasive approach that has shown early promise in alleviating OCD symptoms include transcranial direct current stimulation (tDCS), either increasing (anodal) or decreasing cortical excitability (cathodal). Notably, the right or left OFC has been a chief target for cathodal tDCS whereas anodal tDCS the DLPFC (for a review see, Brunelin et al., 2018).

Moreover, consistent with the CSTC model, a recent study showed decreased activity of the dLPFC during goal‐directed planning in OCD (i.e., in line with prior work; van den Heuvel et al., 2005), as well as decreased functional connectivity between the dLPFC and the basal ganglia. Yet notably, they also found such pattern of aberrant activity in their OCD‐free first‐degree relatives (Vaghi et al., 2017a). In a related vein, Hampshire et al. (2020) found decreased functional connectivity between frontal and occipital cortical areas and the cerebellum during inhibitory control in OCD patients and their asymptomatic first‐degree relatives. Taken together, these results raise the possibility of certain candidate neurocognitive endophenotypes (“vulnerability markers”) for OCD that potentially could help illuminate OC‐related pathology more generally (Hampshire et al., 2020).

Additionally, resting‐state fMRI findings in OCD likewise support the CSTC model. For instance, a meta‐analysis of 541 patients and 572 healthy controls found hypoconnectivity between frontoparietal, default‐mode and salience networks and general dysconnectivity between the frontoparietal network and key CSTC circuits (Gürsel et al., 2018). As such, this provides evidence for the pathological interplay between prefrontal‐parietal‐limbic networks and CSTC loops in OCD (for a related meta‐analysis of resting‐state fMRI overall supporting these findings, see Hao et al., 2019; consistent with the CSTC model, this study consistently found increased ReHo in the lateral OFC in OCD, reflecting neural hyperactivity).

Finally, while research on the neural correlates of the clinical heterogeneity of OCD is scarce, preliminary research indicates the possible involvement of different neural substrates. One study linked the washing and contamination dimension of OCD with reduced gray matter in the bilateral caudate nucleus and white matter volume in the right parietal area. The checking dimension on the other hand, correlated negatively with white matter volume in the temporal lobe; and the symmetry dimension with broader changes such as reduced gray matter volume in the right motor cortex, left insula and parietal region (van den Heuvel et al., 2009). The limited findings (e.g., due to grouping together patients with heterogeneous symptoms) combined with the ample research showing common impairment on frontostratial tasks suggest core neural deficits shared across OCD subtypes (Robbins et al., 2019). Arguably functional brain imaging studies provide a better way to assess neural correlates of OCD subtypes. Indeed, symptom provocation studies in health volunteers and OCD patients have found that distinct OCD symptom dimensions are mediated by differential circuitry (Mataix‐Cols et al., 2003, 2004). For instance, in OCD patients washing‐related anxiety resulted in greater activation than controls in bilateral ventromedial prefrontal regions and caudate nucleus; whereas checking putamen, thalamus, and dorsal cortical regions, and hoarding precentral gyrus and orbitofrontal cortex. Similarly, as noted, OCD commonly cooccurs with other psychiatric disorders such as depression, anxiety disorders, and the OCRDs (e.g., Fineberg et al., 2007). As such, given this clinically heterogonous picture of OCD, it is not always straightforward to draw definite conclusions from imaging studies examining neural correlates of OCD, which often do not factor in comorbidities, that is, group together patients with clinically heterogeneous symptoms. Indeed, overall, the inconsistencies vis‐à‐vis the inclusion of patients with comorbidities and those on medication remain one of the key methodological drawbacks in OCD neuroimaging research.

Relatedly, while there exists a large body of literature on the neural substrate of OCD, there is limited research on the neural basis of OC‐related disorders. Although beyond the scope of this review, it is worth briefly noting that there appears to be some neural overlap with OCD and a number of these conditions. For instance, one study in skin‐picking disorder found functional hypoactivity in a region entailing the bilateral dorsal striatum, bilateral anterior cingulate, and right medial frontal regions—abnormalities chiefly exterior to the usual “dorsal planning network” during an executive planning task (Odlaug et al., 2016). In contrast, OCD circuit abnormalities, as reviewed, include both medial and lateral regions of the prefrontal cortex. These areas involved in skin‐picking disorder are anatomically adjacent to regions also implicated in trichotillomania neuropathology and previous studies of skin‐picking disorder (i.e., MRI research) (e.g., Chamberlain et al., 2010; Grant et al., 2013). In brief, regions mediating these two OC‐related conditions, unlike OCD, thus appear to me more limited to areas mediating habit formation, action monitoring, and top‐down inhibitory control. BDD research on the other hand is mixed with some studies showing structural similarities with OCD; for example, a small study showed caudate volume asymmetry and greater total white matter relative to healthy volunteers (Rauch et al., 2003). Other research has not found volumetric differences in BDD compared to healthy controls (Feusner et al., 2009). Likewise, fMRI research has yielded mixed results. One study, reminiscent of OCD, showed hyperactivy in the left orbitofrontal cortex and bilateral head of the caudate when BDD patients were visually exposed to a photograph of their own face versus a familiar face (Feusner et al., 2010). Unlike OCD however, BDD subjects have been found to show greater left hemisphere activity than controls and abnormal amygdala activation in response to face stimuli (Feusner et al., 2007). (For an overview of the neural overlap between OCD‐ and OC‐related disorders, see Phillips et al., 2010.)

#### 2.2.2 Serotonin hypothesis

A “serotonin hypothesis” implicating the serotonin system in the pathophysiology of the disorder has been posited, arising from the therapeutic effects of the serotonin (5‐HT) reuptake inhibitors (SRIs) on OCD (Barr et al., 1993; Fineberg et al., 2012; Insel et al., 1985; for a recent study on the structure of the serotonin system using positron emission tomography [PET], see also Beliveau et al., 2020). The serotonin (5‐HT) reuptake inhibitors (SRIs) include SSRI drugs (e.g., citalopram, fluoxetine, fluvoxamine, paroxetine, and sertraline) and the tricyclic medication (TCA) drug clomipramine (Dougherty et al., 2004; Fineberg et al., 2015; for meta‐analyses see, Jefferson et al., 1995; Soomro et al., 2008; Stein et al., 1995; for a review, see Fineberg & Gale, 2005). SRI medications inhibit serotonin reuptake at the level of the synapse thereby acutely increasing serotonin availability (Goddard et al., 2008). Although to date there is no overarching model of serotoninergic dysfunction in OCD and the mechanisms whereby SRIs ameliorate symptoms are not well understood (Fineberg et al., 2012; Stein, 2002), suggestions have been made (see also Lissemore et al., 2021).

SRIs might improve OCD symptomatology by modulating orbitofrontal striatal function (Maia & Cano‐Colino, 2015). For example, an early study showed that administration of the SSRI drug paroxetine for 8–12 weeks resulted in reduced activity of the right anterolateral OFC and right caudate nucleus (Saxena et al., 1999). OFC function per se is greatly modulated by serotonin (e.g., Clarke et al., 2004). The OFC is well‐connected to the raphe nucleus, including the dorsal raphe nucleus (Maia & Cano‐Colino, 2015), which may have the greatest number of serotonin neurons in the brain (Liu et al., 2002). One key impairment linked to aberrant OFC activity concerns the ability to appropriately switch behavior to a change in reward and punishment values of stimuli (i.e., a reversal in stimulus‐reward contingencies), so‐called reversal learning (Maia & Cano‐Colino, 2015). Research in primates has shown that selective serotonin depletion of the PFC results in impairments on an OFC‐reliant reversal learning task (Clarke et al., 2004). Another (likewise marmoset) study showed that selective dopamine depletion in the OFC did not lead to such reversal learning deficits, illustrating neurochemical specificity for serotonin (Clarke et al., 2007). Relatedly, research in humans has revealed that acute administration of the SSRI citalopram leads to impairments in reversal learning (Chamberlain et al., 2006b). (Of note, acute SSRI administration is thought to result in serotonin reduction, unlike chronic administration, due to autoreceptor activity. For example, in guinea pigs with a similar subtype of terminal serotonin autoreceptors as humans [Bergqvist et al., 1999], unlike 3 weeks, 8 weeks of the SSRI paroxetine leads to elevated serotonin release in the OFC; owing to desensitization of 5‐HT_1D_ autoreceptors. This mirrors the delayed time course for the effects of SSRIs during OCD treatment [El Mansari et al., 1995; see also, El Mansari & Blier, 2006].) Notably, OCD individuals and their (OCD‐free) first‐degree relatives display decreased activation of the lateral OFC, during a reversal learning task. This suggests that such OFC‐dependent reversal learning‐type hypoactivity constitutes an OCD endophenotype, a brain marker in those with genetic susceptibility (Chamberlain et al., 2008).

Similarly, SRIs may improve structural abnormalities in OCD. For instance, one study found that patients with OCD had greater asymmetry of the amygdala compared with healthy controls prior to pharmaco‐intervention, with a greater left versus right amygdala volume. After treatment with paroxetine however, the left amygdala decreased in volume in OCD patients, unlike healthy controls, and was linked to higher paroxetine dosage during follow‐up (Szeszko et al., 2004). In brief, this study suggests that boosting serotonin may result in reduced amygdala volume in OCD. As will be discussed below, the amygdala has been implicated in the pathogenesis of OCD (e.g., owing to its role in fear conditioning) and is a key site for SRIs to exert their effects. Likewise, another controlled study showed that 12 weeks of SRI (fluoxetine) treatment resulted in greater volume of the left putamen in OCD patients (now comparable to healthy controls) unlike CBT‐treated patients (Hoexter et al., 2012; for a review see, Atmaca, 2013; for a meta‐analysis from the ENIGMA‐OCD consortium showing that SRIs alter brain volumes in OCD, see Bruin et al., 2020).

In cases where OCD patients are unresponsive to such treatment, given for an adequate duration and at an adequate dose, low‐dose antipsychotic agents are sometimes added as an adjunct to SRIs (for a meta‐analysis, see Fineberg et al., 2006; see also Sareen et al., 2004). Antipsychotic drugs are usually potent serotonin 2a receptor (5‐HT_2A_R) antagonists (Fineberg et al., 2006; Marek et al., 2003). Marek et al. (2003) have suggested that the benefits of combining SRIs and antipsychotic drugs may arise from a particular synergistic effect namely: the activation of a number of 5‐HT receptors (i.e., via serotonin reuptake inhibition exerted by SRIs), in conjunction with a specific 5‐HT_2A_ receptor antagonism (i.e., exerted by antipsychotics). This seems to explain the reported beneficial effects of antipsychotics that are strong 5‐HT_2A_ receptor antagonists (e.g., quetiapine and risperidone) but not antipsychotics with higher affinity instead for D_2_ receptors relative to 5‐HT_2A_ (e.g., haloperidol) (Fineberg et al., 2006).

While clinical observation points to the involvement of the 5‐HT_2A_R in OCD, imaging research has also examined these receptors via PET in OCD. For example, Adams et al. (2005) showed that unmedicated OCD patients had enhanced 5‐HT_2A_R binding in the caudate nuclei; and argued that this may possibly be due to the compensatory effects of low availability of serotonin within CSTC circuits. Another study, however, did not find elevated 5‐HT_2A_ binding in the OFC in OCD (also in unmedicated patients), but did show that an earlier onset of the disorder was linked to increased 5‐HT_2A_R availability in this cortical region (Simpson et al., 2011). Taken together, research regarding 5‐HT_2A_R binding is intriguing but inconclusive (Maia & Cano‐Colino, 2015), and the exact role of 5‐HT in OCD remains elusive (Stein, 2002; Westenberg et al., 2007), further complicated by the fact that only around 40–60% of patients improve following SRI intervention (Dougherty et al., 2004; Menzies et al., 2008).

In light of the models of serotoninergic dysfunction in OCD, reviewed above, serotonin system genes have been implicated in OCD pathogenesis. Indeed, research suggests the involvement of genetic risk factors in OCD. For example, the disorder runs in families which for instance is shown in twin studies (Pauls et al., 2014). While research to date is inconclusive, serotonin transporter polymorphism 5HTTLPR and HTR2A are most commonly linked to OCD (Sinopoli et al., 2017).

### 2.3 The role of dopamine

The fact that antipsychotic drugs that modulate dopamine activity may improve OCD symptoms (when combined with SRIs) suggests dopamine involvement in OCD pathophysiology (for reviews, see Denys et al., 2004; Koo et al., 2010). This is consistent with imaging studies in OCD revealing increased dopamine concentrations in the basal ganglia (Denys et al., 2004). For example, one study showed that unmedicated OCD patients had enhanced dopamine transporter binding ratios in the right basal ganglia relative to healthy volunteers (Kim et al., 2003). Relatedly, another study found increased dopamine transporter density in the left caudate and left putamen in unmedicated OCD patients compared to healthy controls, again compatible with CSTC models of OCD pathology (van der Wee et al., 2004). Finally, dopamine agonists acting on the basal ganglia can generate OCD‐like behaviors in both animals (Szechtman et al., 1998), and humans (Borcherding et al., 1990), indicative of a possible role of subcortical dopamine in OCD (see also, Stein, 1996). Cortical dopamine may play a different role in OCD. Enhancement of cortical dopamine using the catechol‐o‐methyl‐transferase (COMT) inhibitor tolcapone significantly improved OCD symptoms, in a 2‐week double‐blind placebo‐controlled study (Grant et al., 2020). Moreover, antipsychotic drugs that block subcortical dopamine receptor activity, as an adjunct to SSRIs, may target the habit system (e.g., damping down aberrant activity) and compulsive behaviors. Antipsychotic medications are used “off‐label,” on an individual patient basis, in some settings to help manage stereotyped behavior in autism spectrum disorder, and self‐injurious behaviors. It is thus plausible that these drugs counteract irrational obsessions or magical ideation in OCD resulting in harmful compulsions such as cleaning hands using bleach (Gillan et al., 2016). This should be explored empirically.

### 2.4 The role of glutamate

Another neurotransmitter implicated in OCD pathology is glutamate, the main excitatory neurotransmitter within CSTC loops (Marinova et al., 2017). Imaging research has shown that OCD patients (i.e., an unmedicated pediatric sample) have raised glutamate concentrations in the caudate relative to healthy individuals. Interestingly, this study showed that caudate glutamate concentrations normalized post 12 weeks of SSRI (paroxetine) treatment (Rosenberg et al., 2000). This suggests that an elevation in serotonin levels may inhibit abnormally raised caudate glutamate activity (Moore et al., 1998). That is, SSRI‐induced serotonin alterations in the frontal cortex may impact cortical‐striatal glutamate projections in OCD (i.e., with great frontocortical glutamatergic innervations to the caudate; Rosenberg et al., 2000), whereas the absence of these inhibitory effects of serotonin within CSTC circuits might allow for elevated glutamate activity within these loops (Goddard et al., 2008). Moreover, in line with the idea of glutamate dysfunction in OCD, some research has revealed increased glutamate concentrations in cerebrospinal fluid (CSF) of (unmedicated) OCD patients (Chakrabarty et al., 2005). Unsurprisingly, given this glutamate imbalance hypothesis, glutamatergic agents have become a focus of interest, particularly for treatment‐resistant OCD. Notwithstanding promising findings (e.g., for glutamate modulators like memantine and n‐acetyl cysteine), this research is still preliminary (Oliver et al., 2015; Pittenger, 2015; see also, Marinova et al., 2017). The glutamate modulator n‐acetyl cysteine has shown evidence of efficacy, in double‐blind placebo‐controlled studies, for OC‐related disorders trichotillomania and skin‐picking disorder, which are OC‐related disorders (Grant et al., 2009; Grant et al., 2016). On the whole, future research will need to shed further light on the role of glutamate in OCD.

### 2.5 Etiology: learning‐based and related cognitive models of OCD

#### 2.5.1 Learning‐based models

Learning‐based models of OCD (derived from Mowrer's two‐process model of fear [1960]) posit that obsessional fear acquisition (via classical conditioning) and extinction are crucial in the etiology and subsequent treatment of OCD (Foa, 2010; McGuire et al., 2016; Shafran, 2005; Taylor et al., 2007; Tracy et al., 1999). Fear and obsessive thoughts are acquired through the pairing of a neutral stimulus (e.g., a doorknob) with a distressing event, for example, contracting a sexually transmitted disease (unconditioned stimulus) after touching a contaminated doorknob (conditioned stimulus) in a public restroom. Later encounters with the conditioned stimulus (doorknob) can now trigger a conditioned response (e.g., excessive contamination concerns) (Foa, 2010; McGuire et al., 2016; Taylor et al., 2007). The individual later learns that repetitive cleansing and avoidance behaviors ameliorate the obsessions and contamination fears. Indeed, these behaviors are negatively reinforced by distress reduction (via operant conditioning); in turn, the obsessional fears are never subject to extinction (Shafran, 2005; Taylor et al., 2007; Tracy et al., 1999). Next, stimulus generalization takes place where the learned fear response is generalized to other stimuli (e.g., a toilet seat), associated with the conditioned stimulus (Dunsmoor et al., 2012; McGuire et al., 2016). These thus become secondary conditioned stimuli (Rachman, 1977). Of interest, anxiety‐related pathology may be characterized by excessive stimulus generalization (McGuire et al., 2016).

While conditioned fear responses are generally robust over time (Butcher et al., 2008), extinction occurs when the individual is repeatedly exposed to the conditioned stimulus (e.g., touching a dirty doorknob) without the aversive outcome (unconditioned stimulus, for example, contracting herpes) and prevention from performing compulsive acts (e.g., excessive handwashing) (McGuire et al., 2016). As a result, the pathological association is degraded or a new nonthreatening stimulus‐response link is established effectively suppressing the previous aberrant association (Jacoby & Abramowitz, 2016; McGuire et al., 2016; Tracy et al., 1999). This model has resulted in the behavioral intervention for OCD called exposure and response prevention (ERP) (Shafran, 2005; Taylor et al., 2007; Tracy et al., 1999). ERP entails exposure to anxiety‐inducing objects and the prevention of ritualistic safety behaviors (Foa, 2010; Shafran, 2005).

### 2.6 Cognitive models of OCD

Mirroring learning‐based theories, according to cognitive models, faulty beliefs give rise to obsessional anxiety. Compulsive acts are then performed to ameliorate intrusive thoughts and anxiety, for example, to ward off aversive outcomes (Abramowitz, 2006; Abramowitz et al., 2005; Gillan & Robbins, 2014). Such cognitive errors include (1) “heightened responsibility”: having a distinct ability to precipitate and/or responsibility to avert aversive outcomes; (2) “overemphasis on thought”: for instance, that merely thinking of an event increases the likelihood of its occurrence; (3) “controlling thoughts”: that full control and regulation of one's thinking is attainable and essential; (4) “overestimation of threat”: that aversive outcomes have a high probability of occurring and disastrous consequences; (5) “perfectionism”: that actions have to be done exactly the “right way,” and overall intolerability toward errors; (6) “intolerance of uncertainty”: for instance, a need to be fully reassured that aversive events will not unfold (Abramowitz, 2006). To target these cognitive biases, “cognitive therapy” was developed (Rosa‐Alcázar et al., 2008). Cognitive therapy typically involves teaching patients about the implausibility and maladaptive nature of these cognitions (i.e., how they give rise to obsessions) (Abramowitz, 2006).

Ostensible strengths of the learning‐based and cognitive models of OCD have been highlighted in the literature. For example, research has shown—as their theories would predict—that exposure to obsession‐related stimuli raises anxiety levels and that subsequent engagement in compulsive rituals lowers such distress, *prima facie* indicating a causal relationship between obsessive ideation and compulsions (Foa, 2010; Shafran, 2005). Another suggested strength of the learning‐based model is the assumption that learning mechanisms driving obsessions and compulsions in OCD are not pathological in themselves. This dovetails with research showing that the vast majority of people in the general population occasionally have obsessive‐like intrusive thoughts that bear resemblance to clinical obsessions (e.g., in terms of content) (Rachman & de Silva, 1978). According to this model, the vast majority of people are not bothered by such intrusions and view them as benign. Indeed, intrusions only become pathological when fear acquisition takes places, that is, when associated with anxiety (i.e., via classical conditioning) (Shafran, 2005). Consistent with this model, as noted, Apergis‐Schoute (2017) showed that OCD patients have difficulties shaking off fear responses when these are no longer threatening; that is, they exhibit impaired safety signaling and that such deficit in safety signaling is mediated by abnormal activity in the salience network, including the ventromedial prefrontal cortex (involved in assigning value to stimuli.) Arguably, however, the most obvious strength is that these models have provided a theoretical framework for widely used psychological therapies for OCD; the learning‐based model, the first‐line nonpharmacological intervention ERP (Shafran, 2005; Taylor et al., 2007; Tracy et al., 1999).

### 2.7 Cognitive deficits in OCD

#### 2.7.1 Cognitive flexibility

Compulsions may be contributed to by impaired “set shifting”—a common cognitive deficit in OCD. Cognitive inflexibility entails reduced ability to appropriately shift attention (e.g., Chamberlain et al., 2005). Notably, research suggests that impaired “set shifting” is a possible endophenotype (Chamberlain et al., 2007b) that is a neuromarker in those with a genetic vulnerability, and a key component of the cognitive profile of OCD (for meta‐analyses, see Abramovitch et al., 2013; Bora, 2020; Snyder et al., 2015) (see also Jalal et al., 2018; Martínez‐Esparza et al., 2021; Rosa‐Alcázar et al., 2021).

The Intradimensional‐Extradimensional Set Shift task (IED) (Downes et al., 1989) of the Cambridge Neuropsychological Test Automated Battery (CANTAB) (e.g., Sahakian & Owen, 1992) is commonly used to assess “set shifting” in OCD (reviewed in Olley et al., 2007). Indeed, research has shown that OCD patients’ performance is impaired on the extradimensional shift (EDS) stage of the IED measure (Chamberlain et al., 2006a; Vaghi et al., 2017b; Veale et al., 1996; Watkins et al., 2005). This ED deficit in OCD has also subsequently been confirmed in meta‐analysis (see Chamberlain et al., 2021).

Cognitive inflexibility in OCD is linked to aberrant processing in frontostriatal circuits, including dorsolateral and ventrolateral prefrontal and striatal areas (Bersani et al., 2013; Isobe et al., 2021; Vaghi et al., 2017b). According to some studies, frontostriatal abnormality may improve in response to treatment in OCD (Freyer  et al., 2011). That is, such studies tentatively suggest that set shifting in particular in OCD may improve following behavioral therapy (Bolton et al., 2000; Katrin Kuelz et al., 2006; Moritz et al., 1999). For example, in Katrin Kuelz et al.’s study (2006), OCD patients displayed greater set shifting deficits at pretreatment than healthy controls but not at posttreatment (i.e., revealing a significant group × time interaction after CBT). As such, set shifting improvements were not solely attributable to practice effects. Yet this study did not have a proper parallel control condition comprised of treated patients, which constitutes a notable limitation. Not all studies suggest that cognitive function can improve after CBT. For instance, one study by Vandborg et al. (2015) examining executive functions in OCD compared to healthy controls did not find CBT to improve cognitive function when correcting for practice effects; however, this study did not include a control group of OCD patients (for a review, see Vandborg et al., 2012). Overall, it remains inclusive whether set shifting may improve following treatment, and more research with parallel control groups of OCD patients is needed.

#### 2.7.2 Visuospatial memory

Memory impairment plays a role in OCD (e.g., Penadés et al., 2005). This is consistent with the clinical observation that OCD patients often complain about forgetting whether they have performed certain actions, resulting in repetitive ritualistic behaviors such as checking and cleaning (Muller & Roberts, 2005). One key example of a memory impairment in OCD is that of nonverbal visuospatial memory, which involves maintaining and processing visual and spatial information (Nikolova & Macken, 2016). Indeed, one meta‐analysis concluded that OCD patients exhibit severe and consistent visuospatial memory impairments compared to healthy controls (Shin et al., 2014; for other meta‐analyses, see Abramovitch et al., 2013; Bora, 2020; see also, Katrin Kuelz et al., 2006; Martínez‐Esparza et al., 2021; Vandborg et al., 2015).

The Paired Associates Learning test (PAL; Sahakian et al., 1988) is a sensitive marker of visuospatial memory, in which participants have to remember the location of various distinct abstract shapes. A number of studies have shown that patients with OCD perform less well than healthy controls on the PAL (Bersani et al., 2013; Gottwald et al., 2018; Morein‐Zamir et al., 2010), indicating possible abnormal involvement of the prefrontal cortex and medial temporal regions. Research has yielded mixed results as to whether visuospatial memory is amenable to nonpharmacological treatment in OCD. One study found that nonverbal visuospatial memory improved in OCD patients after 12 weeks of CBT treatment (Katrin Kuelz et al., 2006). Another study, to the contrary, did not find an effect of CBT on visuospatial memory and concluded that such a cognitive impairment in OCD might be trait‐related as opposed to state‐dependent (Vandborg et al., 2015); however, as this study did not include a parallel control group of OCD patients, these findings should be interpreted cautiously.

#### 2.7.3 Response inhibition

Response inhibition refers to the ability to suppress a prepotent motor response, a neurocognitive domain related to impulsivity (Aron & Poldrack, 2005; Bari & Robbins, 2013). It has been suggested that the intrusive thoughts and repetitive rituals in OCD may reflect an inability to control and inhibit these cognitions and behaviors, and that as such, OCD can be seen as a disorder of cognitive and behavioral inhibitory failures (Chamberlain et al., 2005). Consistent with this view, meta‐analyses have found response inhibition impairments in OCD (e.g., Abramovitch et al., 2013; Lipszyc & Schachar, 2010; Snyder et al., 2015; see also Martínez‐Esparza et al., 2021).

One measure of response inhibition is the Stop Signal Task (SST; Aron et al., 2003). The SST assesses the ability to stop an already triggered motor response. Response inhibition deficits measured on the SST have been reported in OCD patients (Chamberlain et al., 2006a; Penadés et al., 2007; for a recent meta‐analysis, see Bora, 2020), and their first‐degree relatives without the disorder (Bora, 2020; Chamberlain et al., 2007b). Relatedly, as noted, decreased functional connectivity between frontal and occipital cortical areas and the cerebellum during inhibitory control was found in OCD patients and their asymptomatic first‐degree relatives (Hampshire et al., 2020), likewise suggesting that motor disinhibition may be an endophenotypic marker for brain dysfunction in OCD. To date there is little available research on the impact of treatment on response inhibition in OCD (van Velzen et al., 2014). However, one longitudinal study did find that in OCD patients with response inhibition impairments, such deficits were not improved once their OCD symptoms reached remittance, suggesting perhaps this cognitive‐motor impairment may possibly be treatment resistant (Bannon et al., 2006).

#### 2.7.4 Goal‐directed versus habitual control

Compulsions in OCD such as ritualistic handwashing behaviors seem to be disconnected from the overall goal of the activity (hygiene/avoiding contamination). Patients are cognizant that repetitive handwashing makes no sense—is ineffective and excessive—vis‐à‐vis the desired outcome. Yet they are unable to stop (e.g., washing hands until they bleed). Such behaviors appear to have become undesired and insistent habits (Fineberg et al., 2018). Based on this clinical observation, it has been proposed that the stereotyped behaviors seen in OCD may be controlled by habitual brain systems involving cortico‐basal ganglia circuits (Graybiel & Rauch, 2000). Dual‐system theories of instrumental behavior posit two dissociable brain systems influencing actions (Dickinson & Balleine, 1993). Goal‐directed systems drive purposeful and flexible behaviors performed to achieve a certain desired outcome; for instance, washing hands once after using the restroom to avoid contamination. In contrast, washing hands 10 times (inflexibly and thoughtlessly) after using the restroom indicates an automatized response under the influence of habitual neural systems. In the latter case the repetitive handwashing is overall insensitive to a specific outcome (e.g., hygiene) (Fineberg et al., 2018).

Research supports the idea that OCD patients rely more so on habits (automatized behavior) as opposed to goal‐directed behavior compared to healthy individuals. In one study Gillan et al. (2011) using the Fabulous Fruit Game task trained OCD patients to respond to cues by pressing keys on a computer to win points. During a later stage of the task the patients were told that some of the cues were no longer valuable (i.e., did not gain points). Yet, they continued to press the keys in response to the no longer valuable outcomes (referred to as “slips‐of‐action”), indicating a bias toward habitual and automatic responding (for a related study on habit bias in OCD using a shock avoidance paradigm see, Gillan et al., 2014; for a study on functional neuroimaging of habits in OCD, see Gillan et al., 2015; for a review, see Gillan & Robbins, 2014). Research on goal‐directed behavior versus habitual control in OCD is relatively recent. For this reason, to the best of our knowledge, there are no published studies on whether habitual bias in OCD may improve as a result of treatment. It has been suggested that SSRI treatments could reduce habitual bias and associated compulsive behaviors indirectly by ameliorating anxiety and stress that induce them (Gillan et al., 2016). This fits the notion that stress/anxiety promotes excessive habits (Schwabe & Wolf, 2011) underlying compulsive symptoms (Gillan et al., 2011). On the other hand, ERP therapy might through repeated exposure improve habits in OCD by decoupling habitual stimulus‐response associations (for a review see Gillan et al., 2016).

That compulsions in OCD seem to be disconnected from the overall goal of the activity—appear to have become undesired and insistent habits (Fineberg et al., 2018)—challenges learning‐based and related cognitive models of OCD. These assume that obsessions are primary and drive compulsions (secondary epiphenomena) and that these—seemingly purposeful and goal‐directed—acts are performed to ameliorate obsessional distress (Gillan & Robbins, 2014). But such models cannot fully explain the fact that OCD patients, as noted, often are cognizant that compulsive rituals make no sense; that is, they are ineffective and excessive hence the condition is often described as ego‐dystonic (as noted, there are cases where patients do lack insight; Gillan & Robbins, 2014). Indeed, research on habit formation in OCD has shown that anxiety does not appear to be necessary to trigger compulsive‐like behaviors (Gillan et al., 2014) (see also, Gillan et al., 2015; Gillan et al., 2016; Robbins et al., 2019). This indicates the presence of a behavioral deficit that does not rely on obsessional symptoms (Gillan & Robbins, 2014). Similarly, while nonserotonergic anxiolytics like benzodiazepines are used to alleviate anxiety in OCD, they are not a first‐line evidence‐based treatment for core symptoms. And while “pure obsessions” are rare, it has been suggested that mental compulsions like repeated counting may represent nonfully executed behavioral expressions (for a review see, Robbins et al., 2019). (Arguably, however, in principle it may still be possible for conditioning to persist in spite of such lack of insight/goal‐directed action or anxiety; that is, the association between a conditioned and unconditioned stimulus may linger outside the patient's awareness and patients may not report anxiety in association with the conditioned action.) Similarly, other potential pitfalls of learning‐based models specifically include a failure to explain why OCD patients often do not have any memory (history) of pertinent fear conditioning episodes that may account for their obsessional concerns (e.g., illness occurring after contamination) (Jones & Menzies, 1998; Taylor et al., 2007). Also, OCD patients’ obsessions and compulsions can shift over time. A patient at one point might excessively wash after shaking hands, and a few months later, instead, engage in compulsive cleansing of household items (Taylor et al., 2007), which fits the view that these rituals represent undesired habits.

#### 2.7.5 OC subtypes, OC‐related disorders, and comorbidities

There is limited knowledge available on OCD subtypes and the specificity of neuropsychological deficits, owing to the tendency for studies to group together patients of the various variants of OCD. One study found that contamination‐related OCD had greater neuropsychological impairment including in cognitive flexibility and response inhibition relative to healthy controls. The study did not find a link between OC symptom severity and neuropsychological function in this group of washers. Notably, also, patients were drug naïve, and the study controlled for the effects of depression. Indeed, depressive symptoms were not associated with cognitive impairment showing specificity for OCD (Saremi et al., 2017). Other studies have not found such specificity suggesting these neuropsychological impairments represent a transdiagnostic trait. For instance, Moritz et al. (2001) found that while OCD patients with comorbid depressive symptoms displayed cognitive impairment, this was not the case for patients with low depression symptoms relative to healthy controls. Another study assessed response inhibition, using the go‐no‐go task in OCD patients with scrupulousness and contamination‐related symptom dimensions relative to healthy volunteers, and did not find support for any deficits in these two groups; the study likewise controlled for depression effects (Rasmussen et al., 2016). The inconsistent findings reported in the literature are likely attributable to the heterogeneity of OCD symptoms, matching and selection criteria, status of comorbidities and history of medication, as well as the neuropsychological tasks used.

On the other hand, research has shown that patients with OC‐related disorders display some of the same cognitive deficits as those of OCD patients. For instance, one study found that those with BDD performed worse than healthy volunteers on measures of cognitive flexibility and response inhibition, dovetailing with new reclassification of BDD alongside OCD (Jefferies‐Sewell et al., 2017). Another study however found that BDD patients showed impairments in cognitive function but not visual and spatial memory. In this latter study the severity of BDD symptoms, depressive and anxiety symptoms were not associated with cognitive performance (Dunai et al., 2010). Yet another study compared cognitive function in comorbidity‐free OCD and trichotillomania patients and found that while the two disorders entailed spatial working memory deficits, impairments in OCD spanned additional cognitive areas such as executive planning (Chamberlain et al., 2007).

Research has also directly compared the two OC‐related disorders, skin‐picking disorder and trichotillomania, and found that the former was linked with deficits in response inhibition but not cognitive flexibility relative to healthy volunteers; trichotillomania by contrast took an intermediary position vis‐à‐vis response inhibition relative to healthy controls and skin‐picking disorder patients: trichotillomania patients however did not display impairments in cognitive flexibility (Grant et al., 2011). Taken together, the research on cognitive function in OC‐related disorders, while limited, suggests that cognitive impairment in OCD and these disorders share *some* common underlaying neuropathology. That is, for instance, while skin‐picking disorder and trichotillomania both include impairments in motor behavior akin to OCD, patients with OCD display deficits in a wider area of domains including higher‐order cognition, such as, cognitive flexibility, compared to these disorders. Finally, it should be noted that recent research on cognitive function and psychopathology suggest that impairments may cut across multiple disorders, pointing to a single dimensional model of psychopathology. In this model cognitive impairment is considered an inherent construct, that is, a transdiagnostic phenomenon. Indeed, a recent review of meta‐analyses examining cognitive function across all disorders for which studies were available, including OCD, found that all these were linked to cognitive deficits (Abramovitch et al., 2021). Accordingly, it is important that future research on cognitive function in OCD (and OC‐related disorders) includes comorbidity‐free patients (e.g., depression and anxiety) to rule out the confounding effects of comorbid disorders and establish specificity.

## 3 SUMMARY AND CONCLUDING REMARKS

In this review, we provided an overview of OCD symptoms, contemporary diagnostic considerations—for example, vis‐à‐vis the new OCRD classification—and common comorbidities. As seen symptoms can be categorized into partially distinct subtypes, including contamination fears/compulsive cleaning; fears of causing harm/compulsive checking; symmetry obsessions/compulsive ordering; and compulsive hoarding (Abramowitz et al., 2009; Stein, 2002); as noted, hoarding compulsivity may be etiologically distinct from other subtypes of OCD (American Psychiatric Association, 2013; Ayers et al., 2017; Chen et al., 2017; Samuels et al., 2002).

We also shed light on the etiology of OCD, which is still not fully understood, including disease models implicating CSTC circuits in OCD pathogenesis. As reviewed, these circuits may contribute to rigid thoughts and behaviors in turn serving to promulgate OCD symptoms (Chamberlain et al., 2005). Moreover, as discussed, such models have been revised over the years and now adopt a more dynamic view of CSTC circuits, for example, as functionally interacting rather than being fully segregated (Milad & Rauch, 2012; van den Heuvel et al., 2016). Likewise, these models are now more elaborate, for example, consider specific functions of OFC subareas (lateral versus medial) in relation to affective‐, reward‐ and fear‐based computation (Milad & Rauch, 2012; Robbins et al., 2019).

We also discussed the neurochemistry and related pharmacotherapy germane to CSTC loops such as the role of serotonin, dopamine and glutamate systems which remains inconclusive. As noted, the benefits of SRIs might stem from their modulating effects on orbitofrontal striatal function (Maia & Cano‐Colino, 2015), including putatively inhibiting elevated glutamate activity within CSTC circuits (Moore et al., 1998). This may improve the ability to flexibly switch behaviors in response to emotional input (Maia & Cano‐Colino, 2015). Antipsychotic drugs (as an adjunct to SSRIs) that block dopamine receptor activity, may exert their beneficial effects by reducing aberrant dopamine activity within subcortical component of CSTC loops. In contrast, initial research indicates that selective enhancement of cortical dopamine may ameliorate OCD symptoms, according to a double‐blind placebo‐controlled study (Grant et al., 2020).

Moreover, we provided an overview of learning‐based and related cognitive models in the etiology of OCD. As reviewed, a key strength of these models is that they have provided a theoretical framework for widely used psychological therapies for OCD including ERP (the first‐line nonpharmacological treatment) (Shafran, 2005; Taylor et al., 2007; Tracy et al., 1999). But these models assume that obsessions are primary and drive compulsions performed to neutralize obsessional ideation (Gillan & Robbins, 2014), which may not always be the case. Indeed, as noted, arguably such models cannot fully account for compulsions appearing to have become senseless habits for many patients (Fineberg et al., 2018) and that anxiety is not always causal in triggering compulsive‐like behaviors (Gillan & Robbins, 2014; Gillan et al., 2015; Gillan et al., 2014).

As reviewed, key cognitive deficits in OCD include domains such as cognitive flexibility, visuospatial memory, response inhibition, and goal‐directed behavior. As seen, impaired cognitive flexibility may predispose to OCD, and ultimately contribute to the persistence of such symptoms, representing a possible endophenotype. Similarly, intrusive thoughts and repetitive rituals might reflect an inability to inhibit these cognitions and behaviors; indeed, response inhibition may represent another endophenotype for OCD (Chamberlain et al., 2005, 2007b).

While this review provided an overview of key cognitive deficits in OCD, the list is certainly not exhaustive. For instance, other OCD‐related cognitive symptoms highlighted in the literature include greater risk aversion (e.g., repeated handwashing is seen as an attempt to avoid the risk of “contamination”), with mixed empirical support (e.g., Sip et al., 2018; for an overview, see Croft et al., 2022), and excessive performance monitoring, which on the other hand is reliably found in OCD and has been suggested to represent an endophenotype for the disorder (for a review see, Endrass & Ullsperger, 2014). Future comprehensive reviews of cognitive symptoms in OCD should discuss these in more detail.

In conclusion, in this review, we provided an overview of symptoms in OCD and the four partially distinct subtypes. We shed light on the etiology of OCD still not fully understood, including neurobiological theories implicating CSTC circuits and their putative underlying neurochemistry, as well as learning‐based and cognitive etiological models, highlighting their key strengths and weaknesses. Finally, we reviewed major cognitive deficits in OCD affecting domains such as cognitive flexibility, visuospatial memory, response inhibition, and goal‐directed behavior associated with aberrant activity within CSTC loops.

Future research should examine in greater detail how specific CSTC circuits underlie not only clinical symptoms but also cognitive impairments in OCD and the functional interactions between loops. In particular, given the shift in viewing OCD as a “disorder of compulsivity,” research should shed further light on how the “sensorimotor circuit” within CSTC loops mediates habit formation that may promulgate compulsive symptoms. Research should also further disentangle the neurochemistry and related pharmacotherapy relevant to CSTC loops, including the modulating effects of SRIs on orbitofrontal striatal function, and how antipsychotics, as an adjunct to SSRIs, exert their beneficial effects. The glutamate modulator n‐acetyl cysteine shows efficacy for OC‐related disorders, in available controlled studies albeit of small number, and could help shed light on the role of glutamate in OCD. Moreover, research should clarify whether cognitive deficits vary based on OCD subtypes, and whether cognitive impairments are present/differ in childhood/early onset OCD, for example, by analysis of longitudinal data of OCD children and adolescents (for a review, see Marzuki et al., 2020). It would also be useful for future research with proper parallel control groups of OCD patients to examine whether cognitive deficits may improve in response to psychological as well as pharmacological treatment, for example, vis‐à‐vis response inhibition and habit bias, which is lacking.

Finally, this review may help inform diagnosis and treatment of OCD. For instance, we have highlighted that the causal relationship between obsessions and compulsions is not always clear, flying in the face of the traditional view that obsessions lead to compulsions. As work on habit formation in OCD has shown anxiety is not necessary to trigger compulsive‐like behaviors, suggesting that compulsive rituals could represent purely behavioral deficits. We have also reviewed how OCD patients have varying degree of insight into the senselessness of their rituals and that this insight may differ between patients for specific symptoms and can shift over time. Accordingly, clinicians during diagnosis should be cognizant that anxiety is not invariably part of the clinical picture in OCD and such variability of self‐insight. Moreover, given that compulsive rituals in OCD may represent undesired habits, there is a need for early intervention. Stimulus‐response associations, which may underpin compulsions, tend to crystalize by the time patients often receive a diagnosis and initiate treatment. Analogous to other disorders characterized by compulsivity such as addiction OCD becomes harder to treat with time (Gillan et al., 2016). Also, given the role of fear acquisition and extinction in OCD (e.g., that OCD patients exhibit impaired safety signaling), future research should stress tolerable interventions, with a high degree of context specificity, that is, therapeutically beneficial vis‐à‐vis extinction. Indeed, these allow for learning to generalize to real‐world settings where the fear is initially acquired, unlike ERP done in nonnaturalistic environments (i.e., the clinic), where extinction may not fully apply. Finally given the critical role of cognitive inflexibility in OCD thought to reflect repetitive and stereotyped symptoms, future interventions should explore targeting this deficit more directly. As noted, anteromedial subthalamic nucleus DBS in OCD was previously shown to be linked with improved cognitive flexibility (as discussed in a recent review, smartphone interventions may be highly suited for this unmet need; for details, see Jalal et al., 2020).

## FUNDING

This research project did not receive any specific grant from funding agencies in the public, commercial, or not‐for‐profit sectors. SRC's role in this research is funded by a Wellcome Trust Clinical Fellowship (110049/Z/15/Z & 110049/Z/15/A). BJS receives funding from the Wellcome Trust, the Lundbeck Foundation, and the Leverhulme Trust. BJS's research is conducted within the NIHR MedTech and in vitro diagnostic Co‐operative (MIC) and the NIHR Cambridge Biomedical Research Centre (BRC) Mental Health Theme and Neurodegeneration Theme.

## CONFLICT OF INTEREST STATEMENT

BJ has no competing interests to declare. SRC receives honoraria for editorial work at Elsevier in his role as associate editor at NBBR and Comprehensive Psychiatry journals. SRC previously consulted for Promentis (past 3 years). BJS consults for Cambridge Cognition. Authors report no other potential conflicts of interest.

### PEER REVIEW

The peer review history for this article is available at https://publons.com/publon/10.1002/brb3.3000.

## Data Availability

Data sharing is not applicable to this article as no new data were created or analyzed in this study.
